# Real teams and their effect on the quality of care in nursing homes

**DOI:** 10.1186/1472-6963-13-499

**Published:** 2013-12-01

**Authors:** Anders Kvale Havig, Anders Skogstad, Marijke Veenstra, Tor Inge Romøren

**Affiliations:** 1 Norwegian Social Research (NOVA), Oslo, Norway; 2 Centre for Care Research, Gjøvik University College, Gjøvik, Norway; 3 Faculty of Psychology, University of Bergen, Bergen, Norway

**Keywords:** Nursing homes, Team, Teams, Teamwork, Quality of care, Long-term care

## Abstract

**Background:**

Use of teams has shown to be an important factor for organizational performance. However, research has shown that a team has to meet certain criteria and operate in a certain way to realize the potential benefits of team organizing. There are few studies that have examined how teams operate in the nursing home sector and their effect on quality of care. This study investigates the relationship between teams that meet an academic definition of the team concept and quality of care in nursing homes.

**Methods:**

A cross-sectional survey of forty nursing home wards throughout Norway was used to collect the data. Five sources of data were utilized to test our research question: (1) self-report questionnaires to 444 employees, (2) interviews with 40 ward managers, (3) self-report questionnaires to 40 ward managers, (4) telephone interviews with 378 relatives, and (5) 900 hours of field observations. Use of teams in nursing home wards was assessed by field observations and by interviews with ward mangers. Quality of care was assessed by data from surveys and interviews with relatives and staff and through field observations. All data were aggregated to the ward level and two-level analyses were used to assess the relationships.

**Results:**

The multi-level analyses showed that teams – as operationalized in the present study – were significantly positively related to two out of the three quality of care indices when controlled for ward size, days of sick leave and care level. One significant interaction effect was found between teams and days of sick leave, implying that the effect of teams decreased with higher numbers of days of sick leave.

**Conclusions:**

The results suggest that teams are related to higher levels of quality of care in nursing homes. However, the study shows that there is a substantial difference between real, functional teams that meet an academic definition of the concept and quasi teams, the latter having a significantly lower effect on quality of care. Hence, nursing home leaders, directors and ward leaders should be aware of the substantial differences betweens dysfunctional – or quasi – teams and real teams, and encourage the development of real functional teams to take advantage of the potential benefits of team organizing.

## Background

While numerous of studies have investigated the effects of staffing levels and staffing ratios on quality of care in nursing homes [1-3], the number of studies investigating the effects of different ways of organizing care staff within a nursing home ward is relatively limited, both internationally [4,5] and in Norway [6]. Considering that the organization of work is emphasized as an important factor for quality of care [3,7-10], this lack of research is unfortunate and peculiar.

One of the core elements regarding organization of work in nursing homes is whether or not the employees work in teams. Despite this, there is a dearth of studies on teams in nursing homes, particular in Europe [11]. In other research fields, though, there is a vast amount of literature on teams and team performance [12-14]. These studies have shown that there are several potential benefits associated with team organizing, including greater job satisfaction [15], greater commitment [16,17] and greater effectiveness and quality [14,15,18,19]. However, even though researchers agree that teams have the potential to improve organizational performance, there is a consensus that the advantage of teams do not come automatically [12,14,19-22]. Hackman [23], a leading researcher within the field has underlined the importance of real teams for successful teamwork. Hackman’s definition of real teams requires the following criteria: a team task, clear boundaries, specific authority to manage their own work processes and high membership stability [23]. Teams that do not meet these criteria, Hackman [23] argues, are less efficient and less likely to realize the benefits derived from team organizing.

The extensiveness of teams varies considerably both within and between countries. In a Norwegian study, Paulsen *et al*. [24] found that in 31% of the nursing home wards were organized into teams, while in a larger US survey, 16% of care workers in nursing homes reportedly work in teams [7]. The size of nursing home teams may also vary. In Norway, teams generally consist of four to eight direct care workers with responsibility for seven to 12 residents. A nursing home team may be monodisciplinary or multidisciplinary, the latter also called interdisciplinary [25]. Monodisciplinary teams consist of one occupational group, which in nursing homes are mostly comprised of nurse aides [26], while multidisciplinary teams consist of different occupational groups. In Norway, most teams are multidisciplinary in nature, consisting of registered nurses, auxiliary nurses, and to a certain extent, unlicensed workers. Physical therapists, occupational therapists or assistant occupational therapists are normally not included the teams, but organized in their own depart that support the care workers when needed.

There are a variety of different definitions of teams. The one we have adopted is from Guzzo *et al.*[19] and is based on the work of Alderer [27] and Hackman [28]: “… a work group that is made up of individuals who see themselves and who are seen by others as a social entity, who are interdependent because of the task they perform as members of a group, who are embedded in one or more larger social system (organization) and who perform tasks that affect others (such as customers or co-workers)” (p. 308) [19]. This definition of teams implies that there has to be a certain level of co-acting and interdependence among the team members as well as membership stability. A more elaborate discussion of the definition is presented in the Method section.

There are, as mentioned above, few studies of the effects of teams in nursing homes. To identify articles about teams and quality of care we searched in PubMed and Google Scholar and used the following terms: “nursing home”, “long-term care”, “skilled nursing”, “team”, “teamwork”, “primary care” and “work groups”. We also used the snowball approach, whereby the references in relevant articles led us to additional related articles. We found only four studies that systematically had investigated the relationship between teams and quality of care. In the most recent study, Temkin-Greener *et al*. [29] found that the use of teams had a significant positive effect on one out of three quality of care outcomes. The effect was not very strong, however. A total of 7418 direct care workers located in 192 nursing homes in New York participated in the survey study. Goldman [30] conducted a survey study in five continuing care retirement communities and found that a primary nursing approach with the use of teams improved quality of care. The data and the method are not properly described, however. Yeatts *et al.*[26] used an experimental design and found that use of teams improved care procedures, internal coordination and communication and resident care. Ten nursing homes participated in the study: five nursing homes established teams and five nursing homes worked as a control group. The teams consisted of certificated nurses only. Teresi *et al.*[31] also studied the effect of a primary care model. Two nursing homes were included in the study, which used a pre-post matched comparison experimental group design. The study found that several, but not all resident outcomes were improved in those units were the intervention were conducted. The intervention consisted of three elements: consistent assignment, implementation of teams and the inclusion of direct care staff in care planning and improving the internal communication. Thus, it is not possible to isolate the effect of teams itself. One urban (n = 195 residents) and one rural (r = 64) facility participated in the study.

Studies of consistent (also called “primary” and “permanent”) staff-resident assignment and quality of care may also be relevant to the study of teams. Consistent assignment implies that care workers are organized in small work groups, and the similarities are illustrated by Castle’s definition of consistent assignment [32]: “consistent assignment means that the same caregivers are consistently caring for the same residents almost every time they are on duty”. For most practical purposes, this definition is quite similar to our definition of teams, in that a certain group of staff work closely together to accomplish a task and are seen as an entity. A majority of the of the studies of consistent assignment show that consistent staff-resident assignment is related to higher quality of care [4,32-35], though there are exceptions [36]. An interesting result regarding these studies is that the effect of consistent assignment seems to increase with higher levels of staff-resident assignment [32], indicating that high membership stability may be important for the effect.

Some studies, although not testing the direct effects of teams in nursing homes, have still yielded contributions to the research field that are worth mentioning. Rantz *et al.*[10] investigated the relationship between staffing and nursing home quality in 92 US nursing homes and underlined the importance of smaller work groups and team processes to accomplish work in the nursing homes. Other studies that support the use of teams and teamwork for quality of care in nursing homes are Tyler & Parker [11], Dellefield [37], Castle *et al.*[38], Parsons *et al.*[39], Eaton [40] and Bowers *et al.*[41]. Furthermore, there are studies of teams in the hospitals sector – which shares several similarities with the nursing home sector – that support the use of teams for quality of care [22,42-44].

Since there are few studies of teams and quality of care in nursing homes and all the studies are conducted in the US, the effect of teams on quality of care in Norwegian nursing homes is still unknown. The four studies that we have identified indicate that the effect of teams is not very strong; however, all the studies show a positive relationship between use of teams and some quality indicators. In addition, there are several studies that (even if they have not investigated the effect of teams on quality of care directly) suggest that teams are vital for quality in nursing homes. Finally, there are several studies in the hospital sector that supports the use of teams. Therefore, we can hypothesize that the use of teams will be significantly related to higher levels of quality of care. The research question in this paper is if teams – as operationalised in the present study – are related to higher levels of quality of care in Norwegian nursing homes.

## Methods

### Research design

A cross-sectional design was used to collect the data required to test our research question. Five different sources of data were utilized: self-report questionnaires distributed to 444 employees in nursing homes, interviews with and questionnaires to 40 ward managers, a telephone survey with 378 relatives and 900 hours of field observations.

### Sample

One to four wards in 22 nursing homes participated in the study, yielding a total of 40 wards. These facilities were located in towns in eleven medium (6 000 – 20 000) and large-sized (> 20 000) municipalities in seven counties (Finnmark, Nord-Trøndelag, Hordaland, Hedmark, Oslo, Akershus and Aust-Agder) across Norway. The seven counties were selected to achieve geographical spread. Special care units for dementia, short-term units, rehabilitation units and hospice units were excluded, as such wards often have a different structure and relatively more staff than ordinary long-term units. All nursing homes were public and nonprofit in nature, and were owned and run by their local municipalities. The nursing homes ranged in size from 20 to 152 beds, with a mean of 63; the wards ranged in size from 7 to 34 beds, with a mean of 18. The number of staff (full-time equivalents) per ward ranged from 6 to 25, with a mean of 14. Nursing home ward was used as the main measurement unit as both team organization and quality of care may vary significantly from one ward to another within one nursing home.

### Data collection

The first author distributed the questionnaires to the staff personally. All staff who were working in their ward during the three to four days of field observations were asked to complete a questionnaire. Staff who worked night shifts were excluded from the study because their work setting differed substantially from those working day shifts and similarly, staff who had worked less than eight weeks in their ward were excluded due to their lack of experience. Each staff member was offered a token gift (approximate value = 2 USD) along with the questionnaire. The questionnaires were completed anonymously and returned in sealed envelopes in a box located in the wards’ staff room. A total of 444 questionnaires were returned, with a range of 5 to 19 per ward and a mean of 11.4. The response rate from the 40 wards varied from 71% to 100%, with a total response rate of 87%.

Relatives answered a survey by phone interview, also conducted by the first author. Thirty five relatives were excluded due to limited contact with the resident or a complicated relationship between the relative and the resident. A total of 378 relatives agreed in to answer the questions, giving a response rate on 71%.

The first author’s interviews with the 40 ward managers were performed in their offices in the course of the week of field observations. The interviews consisted of semi-structured questions. After the interviews, the ward managers answered a questionnaire consisting of specific questions about the ward.

The first author, with six years experience in nursing homes as an unlicensed worker, conducted the field observations. Each ward was visited and observed for a total of between 20 to 30 hours (within three to four days), depending on its size. A uniform was worn during the visits, and the author participated in the daily activities along with the staff. Both day and evening shifts were observed. During the field observations, notes were taken continuously on a PDA (Pocket PC), and the ward was scored according to predefined categories, as described below. To avoid possible bias by a change in staff behavior during the observations, anonymity was guaranteed to all staff participating in the study. A study by Schnelle *et al.*[45] indicates however that staff behavior is not influenced significantly by field observations.

A written informed consent for participation in the study from the staff and the relatives was not obtained. However, both staff and relatives were informed that it was voluntary to participate in the study and that all data were anonymised. Furthermore, none of the questions were related to patients’ characteristics. Some of the relatives were children of or guardians for the resident, however, all the relatives were older than 21 years.

### Dependent variables

Norway has no national register like the MDS or registers about complaints that are accessible for researchers. Furthermore, the external health inspections are randomly conducted and not suited for comparing quality of care between different nursing home wards. Consequently, there is no appropriate register for this data in Norway, implying that researchers must collect quality data themselves.

In the Norwegian long-term care sector Q*uality of care* is regulated by *The National Regulation for Quality of Care in Nursing Homes and Home Care*[46]*.* The regulation has been the starting point for assessing quality of care in several studies [24,47,48]. According to the regulation quality of care is a multidimensional phenomenon, consisting of both quality of care and quality of life aspects. Based on the regulation, and prior studies of quality of care in Norway, we developed four quality dimensions. The dimensions were *medical care, general care, social activities within the ward* and *social interactions between staff and residents* (see Additional file 1: Appendix for details)*.* In addition, we included a general dimension assessing the overall perception of the quality level; “All in all, how do you assess the quality of care at this nursing home ward?”. Thus, in total, five quality dimensions were utilized in the present study (see Table 1 for details about the different dimensions). The dimensions were solely process and outcome measures [49], with an emphasis on outcome measures. Each dimension was measured by one to five items (see Additional file 1: Appendix for an overview of the items used). Staff assessed nine items, relatives eight items and the field observer seven items. All items from relatives, staff and field observations were measured on a Likert scale ranging from one to seven, with 1 anchored at *strongly disagree* and 7 anchored at *strongly agree*. The items were tested on pilot groups of staff, residents and fellow researchers prior to the study, and minor adjustments were made based on the response from these groups.

**Table 1:** Descriptive statistics (N = 40 wards)

**Dependent variables/quality of care (scale 1–7)**			
	**Mean real teams (N = 19)**	**Mean no teams or quasi teams (N = 21)**	**T-test independent samples**
**Relatives** (Cronbach’s alpha = 0.92)			
Medical care	5.45	5.11	(*p* = 0.11)
General care	6.10	5.37	(*p <* 0.01)
Social activities	4.30	3.57	(*p <* 0.03)
Social interactions	5.79	4.98	(*p <* 0.01)
General perception of quality of care	5.83	4.92	(*p <* 0.01)
**Summary index relatives**	**5.49**	**4.79**	(*p <* 0.01)
**Staff** (Cronbach’s alpha = 0.85)			
Medical care	6.09	5.55	(*p <* 0.01)
General care	5.47	5.18	(*p* = 0.08)
Social activities	4.29	4.15	(*p* = 0.64)
Social interactions	5.31	4.45	(*p <* 0.01)
General perception of quality of care	5.95	5.07	(*p <* 0.01)
**Summary index staff**	**5.42**	**4.88**	(*p <* 0.01)
**Field observations** (Cronbach’s alpha = 0.92)			
General care	6.05	5.29	(*p <* 0.01)
Social activities	4.68	3.71	(*p <* 0.01)
Social interactions	5.29	4.19	(*p <* 0.01)
General perception of quality of care	5.53	4.52	(*p <* 0.01)
**Summary index field observations**	**5.39**	**4.43**	(*p <* 0.01)
**Control variables**			
Residents per ward	13.6	22.2	(*p <* 0.01)
Days of sick	11.1%	12.9%	(*p* = 0.26)
Care level			
Level of residents using wheel chair (1–7)†	3.89	5.47	(*p <* 0.01)
level of residents using elevator during care (1–7)††	3.74	5.05	(*p =* 0.26)
**Sum care level (1–7)**	**3.82**	**5.26**	(*p =* 0.01)

† = (1 = < 5%, 2 = 5% to 10%, 3 = 10% to 20%, 4 = 20% to 30%, 5 = 30% to 40%, 6 = 40% to 50% and 7 > 50%).

†† = (1 = < 5%, 2 = 5% to 10%, 3 = 10% to 15%, 4 = 15% to 20%, 5 = 20% to 25%, 6 = 25% to 30% and 7 > 30%).

The responses from relatives, staff and the field observer formed three separate summary indices (see Table 1 and Additional file 1: Appendix). The indices were created by adding the dimensions and calculating the mean value. The indices based on relatives and staff contained all five quality dimensions, while the index based on field observations did not include *medical care*, as the field observer has no medical education and field observations alone did not the put the field observer in a position to assess the medical care satisfactorily. The responses from relatives and staff were aggregated to a ward level. Internal consistency of the indices was high, with Cronbach's alphas of 0.92 for relatives, 0.85 for staff and 0.92 for field observations, and supported the use of summary indices. Factor analysis (using the Varimax method of rotation) showed that the three sources evaluated the quality of care significantly different – in accordance with prior studies of proxies and quality of care in nursing homes [48,50,51] (see Additional file 1: Appendix). The inter-rater correlation coefficient between the three quality indices was 0.63.

### Explanatory variables

To define a team, we used the above definition by Guzzo *et al.*: [19]: “… a work group that is made up of individuals who see themselves and who are seen by others as a social entity, who are interdependent because of the task they perform as members of a group, who are embedded in one or more larger social system (organization) and who perform tasks that affect others (such as customers or co-workers)” (p. 308) [19]. The definition implies that in order to be labelled a team, the work group has to function in a particular way; it is not sufficient that the manager or the care workers label the work group a team. Three different factors were of particular interest when we made the division [19,21,23]. First, we noted the degree of interdependence among the staff members within a subunit: did the care workers within a subunit collaborate closely to accomplish the tasks or were they simply co-acting without being interdependent and well coordinated? Second, we noted the degree of membership stability within the team: did the care workers work on the same subunit every day they were on duty or did they rotate between the different subunits within the ward? Third, we noted whether the staff consisted of a cohesive group that belonged primarily to the subunit or the ward level (in the definition labelled as “larger social system or organization”) and at which of the two levels the primary tasks were executed: did the care workers within a subunit appear like a social entity or did they assess the ward as their main social entity? All three factors had to be fulfilled in order to be labelled a team. Work groups that did not meet the criteria, were defined as dysfunctional – or quasi – teams.

In prior survey studies of teams and teamwork in nursing homes, the assessments of teams were carried out by staff members, ward managers or other stakeholders. Such subjective assessments are vulnerable to individual interpretations of the team concept, and may not systematically differentiate between teams and unstable work groups that do not meet a standardized, academic definition of the concept. Therefore, to account for the potential bias in the team concept, we based the assessment of teams on two different sources: (1) a third part, by conducting three to four days of field observations in each of the participating nursing home wards and (2) interviews with the ward leaders. The field observations lasted three to four days (20 to 25 hours) and consisted of direct observations of the organizational structure and informal interactions with care workers. The interviews of the ward leaders took place at the ward leaders’ office during the visit. When divergence was found between the information gained from the ward leader and the field observations, the observations were decisive – as information obtained through interviews with the ward leader was more likely to be biased by the individual leader’s interpretations of the team concept. Furthermore, in contrast to the ward leaders, the field observer visited all 40 wards and – as a third party – was able to compare the organizational structure between the wards and thus assess the team variable in relation to our standardized definition of teams.

The wards were categorized into two groups (dichotomous variable): Group 1 represented no use of teams or use of dysfunctional teams (i.e., teams that were labelled as teams by staff or managers, but did not actually operate as teams according to our definition), while Group 2 represented use of teams according to our standardized definition. 19 wards operated with teams, 18 operated with dysfunctional teams and three wards reported that they did not use team organizing at all (see Table 1). This implies that 19 wards were categorized as having teams and 21 wards were categorized as not having teams. All the 19 identified teams were multidisciplinary in nature, meaning that they consisted of registered nurses, auxiliary nurses and to a certain extent unlicensed workers. Physical therapists, occupational therapists or assistant occupational therapists were not included in any of the teams.

From earlier studies in nursing homes, we know that organizational characteristics like ownership status, staffing levels, ratio of registered nurses, ward size, care level and staff stability/days of sick leave have an effect on of quality of care [10,47,48,50-54]. Due to our limited sample size (N = 40), we included only three control variables in the regression analyses. Ownership status was not relevant as all nursing homes included in the study were nonprofit and owned and run by the local municipality. Ward size, days of sick leave and care level were the three variables that had the strongest correlations with the three quality indices (see Table 2). Staffing levels and the ratio of registered nurses to residents were thus excluded from our regression model (see Table 2). Information about the confounding variables of *residents per ward* and *days of sick leave* (annual) were obtained through interviews with the ward managers, while the confounders of *care level* was measured by two factors: *the percentage of residents dependent on wheel chair* and *the percentage of residents dependent on patient lift during care*, each of which was allocated a score from 1 to 7 (see Table 1). The care level data were obtained through field observations and interviews with care workers.

**Table 2:** Bivariate correlations (Pearson’s) (N = 40 wards)

	**QoC - relatives**	**QoC - staff**	**QoC - field obser.**	**Team**	**Days of sick leave**	**Ward size**	**Care level**	**Total staffing levels**	**Ratio of registered nurses**
QoC - relatives	1								
QoC - staff	0.63**	1							
QoC - field observations	0.63**	0.65**	1						
Team	0.52**	0.48**	0.70**	1					
Days of sick leave	0.57**	0.71**	0.52**	−0.18	1				
Residents per ward	−0.34*	−0.24	−0.32*	−0.46**	−0.03	1			
Care level	−0.36*	−0.25	−0.22	−0.39*	−0.27	0.50**	1		
Total staffing levels	0.12	0.09	.025	0.26	0.41**	−0.14	−0.65**	1	
Ratio of registered nurses	0.75	−0.05	0.06	0.15	−0.36*	0.09	0.26	−0.14	1

*(*p* < 0.05), ** (*p* < 0.01).

### Data analysis

Mean scores for the three quality of care indices were calculated and independent-samples T-tests were conducted to assess differences between the 19 wards with real teams and the 21 wards with no teams or quasi teams (Table 1). To assess potential multicollinearity problems due to high correlations (>0.80) between the independent variables, we also computed Pearson’s bivariate correlations (Table 2). The associations between teams and each of the three quality indices (relatives, staff and field observations) were assessed with multilevel regression analyses (Table 3). As the 40 nursing home wards were located in 22 different nursing homes, we performed two-level analyses to account for potential clustering effects (random intercept models) [55]. Potential interaction effects between the team variable and each of the control variables were also explored separately (Table 4). In studying interaction effects we centered days of sick leave around the mean, that is: we subtracted the mean numbers of days of sick leave in the sample from each data-point [56].

**Table 3:** Two-level analysis for quality of care as assessed by relatives, staff and field observations: unstandardized coefficients and explained variance - (N = 40 wards and 22 nursing homes)

	**Quality of care - relatives**		**Quality of care - staff**		**Quality of care - field observations**	
	**Coeff.**	***p*-value**	**Coeff.**	***p*-value**	**Coeff.**	***p*-value**
Team	0.347	= 0.08	0.389	= 0.03	0.926	< 0.01
Residents per ward	0.003	= 0.80	0.001	= 0.90	0.002	= 0.88
Days of sick leave	−4.008	= 0.04	−3.854	= 0.02	−0.098	= 0.95
Care level	−0.117	= 0.03	−0.067	= 0.19	0.008	= 0.87
R_1_^2^	0.38		0.15		0.49	

**Table 4:** Two-level analysis with interaction effects for quality of care as assessed by relatives, staff and field observations: unstandardized coefficients and explained variance - (N = 40 wards and 22 nursing homes)

	**Quality of care - relatives**		**Quality of care - staff**		**Quality of care - field observations**	
	**Coeff.**	***p*-value**	**Coeff.**	***p*-value**	**Coeff.**	***p*-value**
Team	0.247	= 0.17	0.299	= 0.09	0.870	< 0.01
Residents per ward	−0.003	= 0.75	−0.002	= 0.79	−0.003	= 0.72
Days of sick leave	−1.479	= 0.44	−2.176	= 0.20	1.679	= 0.38
Care level	−0.125	= 0.01	−0.071	= 0.14	0.003	= 0.93
Team*Days of sick	−12.290	< 0.01	−8.571	= 0.02	−9.033	= 0.02
R_1_^2^	0.50		0.33		0.56	

Data were analyzed using SPSS (Statistical Program for Social Science) version 19 and mixed models were used for the analyses. For all statistical tests a 5% significance level was employed. P-P plots and residual plots were examined prior to the analyses and revealed no violations of assumptions of normality, linearity and homoscedasticity.

In the present paper we only report the results from the two-level analyses, as these analyses are available for all three data sources; relatives, staff and field observations (Tables 3 and 4). However, we also conducted three-level analyses for the quality indices for relatives and staff since we had individual, ward level and nursing home data for these two sources. These three-level analysis yielded similar results and the same conclusions as the two-level analysis though (Additional file 1: Appendix). Also the results of ordinary one-level regression analyses were similar to those of the two-level analyses (data available on request).

### Ethical considerations

The study has been approved by the Norwegian Social Science Data Services (NSD), an institution that assists and approves researchers with data gathering, data analysis, privacy issues and research ethics. All data in the study were anonymous and no separate data about any residents were collected. Consent procedures for this study were approved by staff and residents. Consent procedures included a description of the study, expectations of participation, procedures taken to ensure confidentiality and the voluntary nature of the study. Nursing home staff were provided this information in written format prior to giving verbal consent, while family members were informed over the telephone prior to providing verbal consent. Participants were informed that confidentiality was assured and that they had the right to withdraw from the study at any point. Prior to field observations at the nursing homes, the first author made a declaration of nondisclosure of confidential information.

## Results

Table 1 shows mean scores for the 19 wards with real teams and the 21 wards with no teams or dysfunctional teams and the independent-samples T-tests. The T-test shows that the wards with teams had significantly higher levels of quality of care on 11 out of the 14 quality dimensions and on all three summary indices. Only the dimension of medical care (*relatives*), general care (*staff*) and social activities (*staff*) were not significantly higher in wards with teams. Bivariate correlations are presented in Table 2. The correlations between the explanatory variables were low to moderate (*r* ≤ 0.50), while the correlations between the team variable and the three quality indices were relatively strong (*r* = 0.52) for relatives, (*r* = 0.48) for staff and (*r* = 0.69) for field observations. The wards with teams had significantly lower care level than those with no teams.

The results from the multilevel regression analyses for each of the three quality indices are presented in Table 3. The intra-class correlation coefficients (ICC) for the three indices were 57% (relatives), 20% (staff) and 64% (field observations), respectively. The analyses showed that the team variable was significantly positively related to the quality index by staff (*p* = 0.03) and field observations (*p* < 0.01), but not to the index by relatives (*p* = 0.08) when controlled for ward size, days of sick leave and care level (Table 3). The association between teams and quality as assessed by field observations was particularly strong: wards with teams had 0.93 points higher levels of quality of care than the wards without teams. In the model with staff assessed quality of care the unstandardized regression coefficient was 0.39.

Regarding the control variables, residents per ward was not significantly related to any of the quality indices, care level was significantly negatively related to the quality index by relatives (*p* = 0.03) while days of sick leave was significantly negatively related to the indices by relatives (*p* = 0.04) and staff (*p* = 0.02).

**Figure 1:**
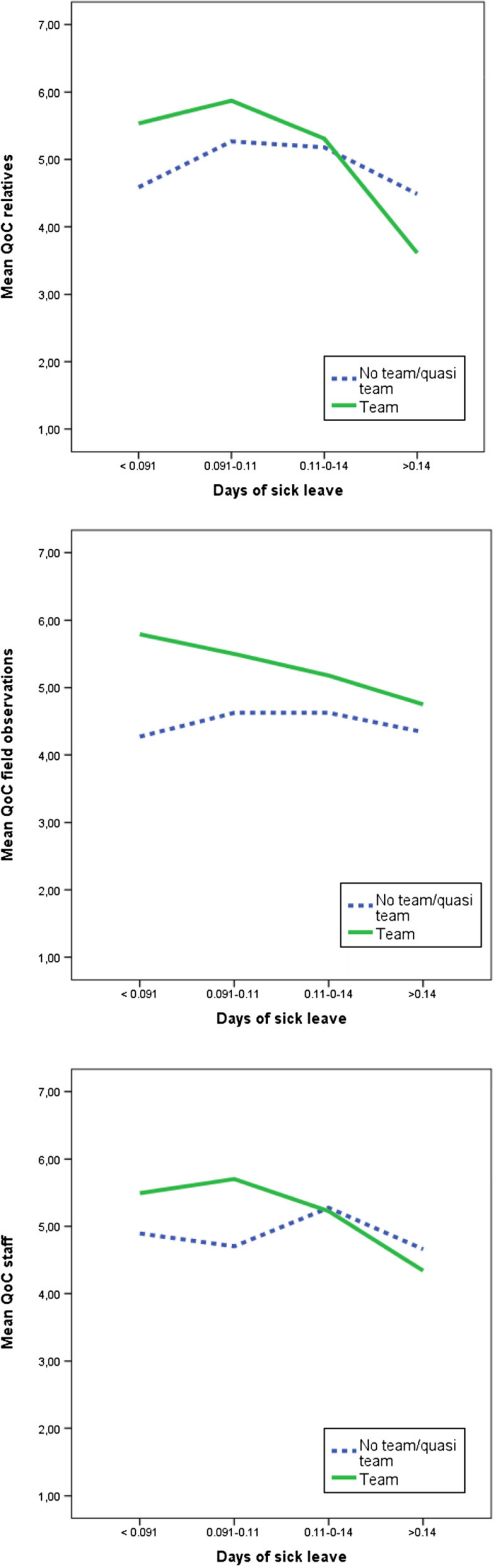
Interaction effects between team and days of sick leave (annual) (N = 40 wards).

One significant interaction effect was found between the team variable and days of sick leave (Table 4 and Figure 1), implying that the positive effect of teams decreased with high rates of days of sick leave. The interaction variable between teams and days of sick leave had a significant effect on all three quality indices, and improved the models significantly.

## Discussion

### Main results

The results showed that teams was significantly positively related to two out of three quality of care indices when controlling for ward size, days of sick leave and care level. Furthermore, the team variable interacted significantly with days of sick leave, implying that the effect of teams decreased with more days of sick leave. Teams was particularly strong related to the quality index based on field observations, as wards that were organized in teams had 0.93 points higher levels of quality than wards that were not organized in teams. Wards that were organized in teams had a significantly lower care level than wards that were not organized in teams. One possible explanation for this is that that wards leaders in wards with high care levels use staff rotation to reduce the wear and tear and boredom of the care workers [4].

The positive effect for teams in our study confirms prior studies of teams and quality of care in nursing homes. However, this study deviates from the prior studies in that the relationship between teams and quality of care were stronger. Hence, the relatively strong support for teams in our study calls for further explanations. In the following section we will argue that there are two factors that contributed to explain the relatively strong effect for teams in the present study; (1) the importance of real teams and (2) the particularity of the Norwegian context.

### The importance of real teams

In prior survey studies of teams in nursing homes, the assessment of teams has been based on self-reported data from the care workers [29,30]. Using self-reported survey data is time-efficient, but vulnerable to individual interpretations of the team concept. To avoid such individual interpretations, the present study based the assessment of teams on two different sources; (1) three to four days of field observations in each of the participating nursing home wards and (2) interviews with the ward managers. By combining these two data sources, we believe that we were able to differentiate more systematically between wards that operated with real, functional teams – teams that met our standardized, academic definition of the concept – and dysfunctional teams – teams that were labelled a team by staff and ward leaders, but did not operate as teams according to our definition – than studies that assess teams solely on survey data from the care workers.

There were mainly two factors that distinguished dysfunctional teams from real teams in our study: First, the staff in the dysfunctional teams – to a considerable extent – rotated among the different subunits in the ward and thus did not meet our criterion of *membership stability*. Second, the primary tasks were executed at the ward level and thus did not meet our criterion of *primary tasks at the subunit*. We believe that both of these criteria are important for team performance [23]. The first criterion, *membership stability*, may – under certain circumstances – be negative for team performance. There is a risk that very stable teams, which are not exposed to new routines, tasks and changes in the environment, will develop unfavorable working environments [57,58]. Yet, in general, team stability has shown to be an important and necessary element for successful teams [14,23,57,59-61]. In line with this, Hackman [23] argues that workers in teams with high membership stability are more likely to develop familiarity with each other and the work setting, and that this familiarity enables the team members to focus on the work tasks rather than to spend time on getting to know new co-workers and include them in the work procedures.. Furthermore, the importance of membership stability for quality of care is supported by the interaction effect that we found between teams and days of sick leave, showing that the positive effect of teams decreased with higher levels of days of sick leave. High levels of days of sick leave result in more vacant and temporary positions, and thus lower the team’s membership stability. Membership stability is also likely to be important for developing a good team climate, particularly the climate factors of interaction and information sharing.

The second criterion, *primary tasks at the subunit* implies that the daily tasks are performed at the team/subunit level and not at the ward level. If there are unclear boundaries between the two levels, the team may not operate as a bounded unit [19,23]. The result may be that the workers experience that their membership status is uncertain, their ability to develop specialized roles, shared norms and a common commitment are weakened and their ability to develop a coherent strategy to carry out the work is limited [21,23,62]. Consequently, they will act more like co-actors and less like team members that are performing teamwork [23,63]. Unclear boundaries may also decrease a team’s ability to develop ownership to the organization and its goals [16,17,64] and to develop a good team climate.

Both the criteria of *membership stability* and *primary tasks at the subunit level* refer to how the team actually operates. The focal point here is that there is a *real, functional team* and not an unstable group of employees who is simply co-acting.

Based on the above discussion, we propose that: (1) real teams, according to our definition and operationalization, is a prerequisite for effective teamwork in nursing homes and (2) that our thorough assessment method enabled us to systematically differentiate between real teams – that meet the criteria of an academic definition of the team concept – and dysfunctional teams. In line with this, we argue that the differentiation between real teams and dysfunctional teams in the present study helps explain the relatively strong effect for teams on quality of care as compared to prior survey studies of teams in nursing homes. We believe that future studies of teams in nursing homes, and in the care sector as a whole, should attempt to distinguish real teams from dysfunctional teams. Furthermore, leaders in nursing homes should be aware of the characteristics that distinguish real, functional teams from dysfunctional teams – in order to take advantage of the potential benefits derived from team organizing [21,23,44].

### The particularity of the Norwegian context

Another factor that may partly explain the positive effect of real teams on quality of care in the present study is the characteristics of Norwegian nursing home teams. Two variables are of particular importance: First, in contrast to the US and several other Western countries where nursing home teams generally consist of nurse aides only [4,26], the majority of Norwegian nursing homes teams are multidisciplinary. This means that the teams consist of registered nurses, auxiliary nurses and to a limited extent unlicensed workers. Second, the hierarchical structure and role pattern in Norwegian nursing homes deviate from many other countries [65-67]. For example, registered nurses usually participate in the daily care tasks along with the other care staff, and are, to a certain degree, equal members of the care teams with the other team members [65-70]. Thus, Norwegian nursing home teams are more egalitarian than teams in most other countries.

In prior research in the health care sector, multidisciplinary teams with low hierarchical relationships have shown to increase team performance [71]. Hence, we propose that these two specific attributes of Norwegian nursing homes teams may contribute to explain the strong relationship between teams and quality of care in the present study, and that these variables should be investigated in cross-national studies in the future.

### Limitations

There are several limitations to our study. First, the number of participating nursing home wards is limited, which implies that our sample is not representative of the population of Norwegian nursing homes. Second, the study had a cross-sectional design. A longitudinal design could have strengthened the conclusions of the study. Third, study findings would have been strengthened with the use of advanced measures for the medical aspects of quality of care, like MDS-data, data from inspections, or data about complaints. However, Norway has no national register like the MDS or any register about complaints, and the external health inspections are randomly conducted and not suited for comparing quality of care between different nursing home wards. Finally, better data about care level would have enabled us to conduct a more advanced case mix adjustment. Unfortunately, there is no national data register of the resident’s health and function level that is easily accessible for researchers. The collection of this data was further complicated by Norway’s strict privacy regulations limiting the collection of individual data about residents.

## Conclusions

Our study results indicate that the teams could improve quality of care in Norwegian nursing homes. Several work groups that labelled themselves as teams did, however, not function as real teams according to our definition. Our analyses showed that these teams – labelled dysfunctional teams – were significantly less effective than those work groups that operated as real teams according to our criteria. Consequently, we argue that simply creating teams in itself is likely not sufficient to improve quality of care; the focal point is that there is a real team performing genuine teamwork. Furthermore, we argue that two factors are particularly important in the creation of real teams within nursing homes: (1) high membership stability and (2) that the main tasks are conducted at the subunit level and not at the ward level. From this finding, we suggest that nursing home leaders, directors and ward leaders should be aware of the substantial differences between dysfunctional teams and real teams; and should support the development of real teams within their wards, which, our study finds, will improve the quality of care for their nursing home residents.

## Competing interests

The authors declare that they have no competing interests.

## Authors’ contributions

AKH participated in the study design, carried out the data collection, performed the statistical analysis and drafted the manuscript. ASK helped to draft the manuscript. MVE participated in the statistical analysis. TIR participated in the study design. All authors read and approved the final manuscript.

## Pre-publication history

The pre-publication history for this paper can be accessed here:

http://www.biomedcentral.com/1472-6963/13/499/prepub

## Supplementary Material

Additional file 1 Appendix. Click here for file
