# Supplementary material for: Real teams and their effect on the quality of care in nursing homes
Source: BMC Health Serv Res. 2013 Dec 1;13:499. doi: 10.1186/1472-6963-13-499 (PMC4220624; doi:10.1186/1472-6963-13-499)
Supplement: Additional file 1 — Appendix. [file 1472-6963-13-499-S1.doc]

**ADDITIONAL FILE 1: Appendix**

**Quality of care items – assessed by relatives, care workers and field observations**

| **Quality of care items (N = 40 wards)** |
| --- |
| **Medical care (1 - 7)** |
| How satisfied are you with the medical care at the nursing home? *(relatives)* |
| The ward has relevant procedures in relation to death *(staff)* |
| The ward gives good care to terminal residents *(staff)* |
| There is a considerable professional medical focus at the ward *(staff)* |
| All pressures ulcer are treated adequately and promptly *(staff)* |
| **General care (1 - 7)** |
| How satisfied are you with the daily care at the nursing home? *(relatives)* |
| How satisfied are you with the personal hygiene at the nursing home? *(relatives)* |
| Do the staff act with dignity and respect during care? *(relatives)* |
| The staff note all relevant care information in the journal *(staff)* |
| The resident get their teeth brushed every day *(staff)* |
| Respect for residents during care activities *(field observations)* |
| Social atmosphere between residents and staff during care *(field observations)* |
| **Social activities within the ward (1 - 7)** |
| How satisfied are you with the level of activities offered to the residents? *(relatives)* |
| The residents are offered a sufficient level of daily activities *(staff)* |
| General level of activities for the residents *(field observations)* |
| The staff arrange coffee breaks etc *(field observations)* |
| **Social interactions between staff and residents (1 - 7)** |
| How satisfied are you with the staff's behavior towards the residents *(relatives)* |
| Do the staff carry conversations with the residents? *(relatives)* |
| Do the staff carry conversations with the residents *(staff)* |
| To what degree do the staff carry conversations with the residents? *(field observations)* |
| Empathy showed by the staff towards the residents *(field observations)* |
| **General perception (1 - 7)** |
| All in all. how satisfied are you with the care at the nursing home *(relatives)* |
| All in all. how would you assess the care the residents receive at this nursing home *(staff)* |
| General assessment of the care at the nursing home *(field observations)* |

**Factor analysis quality of care items – assessed by relatives, care workers and field observations**

| **Factor analysis - Rotated Component Matrix** | | | |
| --- | --- | --- | --- |
|  | Component | | |
|  | **1** | **2** | **3** |
| Medical care - relatives | **0.853** | 0.102 | 0.102 |
| General perception - relatives | **0.828** | 0.409 | 0.141 |
| Social interactions - relatives | **0.799** | 0.372 | 0.280 |
| Social activities - relatives | **0.785** | 0.188 | 0.131 |
| General care - relatives | **0.778** | 0.431 | 0.253 |
| Social interactions - field observations | 0.290 | **0.871** | 0.200 |
| General perception - field observations | 0.266 | **0.861** | 0.200 |
| Social activities - field observations | 0.384 | **0.771** | 0.119 |
| General care - field observations | 0.160 | **0.738** | 0.402 |
| Social activities - staff | 0.251 | 0.017 | **0.760** |
| General care - staff | 0.127 | 0.290 | **0.747** |
| Social interactions - staff | 0.527 | 0.280 | **0.667** |
| General perception - staff | 0.419 | 0.471 | **0.631** |
| Medical care - staff | 0.448 | 0.439 | **0.589** |

**Analysis at individual level (relatives and care workers) and ward level**

| **Two-level analysis for quality of care as assessed by relatives (N = 378) and care workers (N = 444)** | | | | |
| --- | --- | --- | --- | --- |
|  | **Quality of care – relatives**  **N = (40 wards & 378 relatives)** | | **Quality of care – staff**  **N = (40 wards & 444 care workers** | |
|  | Coeff. | *p*-value | Coeff. | *p*-value |
| Team | 0.513 | *p* < 0.01 | 0.427 | *p* < 0.01 |
| Residents per ward | -0.001 | = 0.99 | 0.001 | = 0.91 |
| Days of sick | -4.357 | = 0.02 | -3.299 | = 0.02 |
| Care level | -0.101 | = 0.07 | -0.026 | = 0.51 |

**FIGURES**

**Figure 1 Interaction effects between team and days of sick leave (annual) (N = 40 wards)**
